# Supplementary material for: RHD6LA regulates root hair responses to both symbionts and commensals
Source: Nat Commun. 2026 Mar 10;17:4447. doi: 10.1038/s41467-026-70504-1 (PMC13183932; doi:10.1038/s41467-026-70504-1)
Supplement: Supplementary file 1 — Supplementary Information [file 41467_2026_70504_MOESM1_ESM.pdf]

Supplementary Information

a

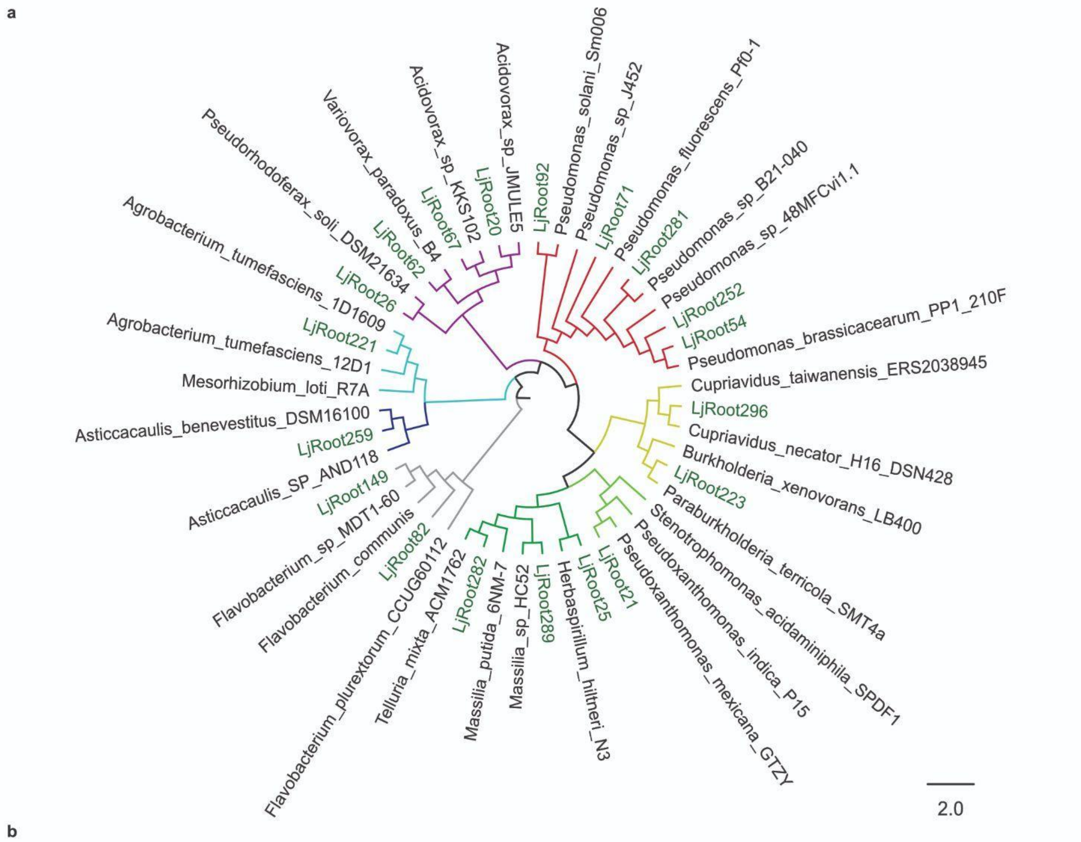

b

Isolate name	Family	Genus	Species (>95% ANI)	Closest reference (>90% ANI)
LjRoot223	Burkholderiaceae	Paraburkholderia	*P. terricola*	*P. terricola SMT4a*
LjRoot296	Burkholderiaceae	Cupriavidus	*C. necator*	*C. necator H16*
LjRoot259	Caulobacteraceae	Asticcacaulis	-	-
LjRoot20	Comamonadaceae	Acidovorax	-	*A. sp. JMULE5*
LjRoot26	Comamonadaceae	Pseudorhodoferax	-	*P. soli DSM21634*
LjRoot62	Comamonadaceae	Variovorax	-	*V. paradoxus B4*
LjRoot67	Comamonadaceae	Acidovorax	-	-
LjRoot149	Flavobacteriaceae	Flavobacterium	-	-
LjRoot82	Flavobacteriaceae	Flavobacterium	-	*F. plurextorum CCUG 60112*
LjRoot25	Oxalobacteraceae	Herbaspirillum	-	*H. hiltneri N3*
LjRoot282	Oxalobacteraceae	Telluria	-	*Telluria mixta ACM1762*
LjRoot289	Oxalobacteraceae	Massilia	-	-
LjRoot252	Pseudomonadaceae	Pseudomonas	*P. brassicacearum*	*P. brassicacearum PP1_210F*
LjRoot281	Pseudomonadaceae	Pseudomonas	*P. sp. B21-040*	*P. sp. B21-040*
LjRoot54	Pseudomonadaceae	Pseudomonas	*P. brassicacearum*	*P. brassicacearum PP1_210F*
LjRoot71	Pseudomonadaceae	Pseudomonas	-	-
LjRoot92	Pseudomonadaceae	Pseudomonas	*P. solani*	*P. solani Sm006*
LjRoot221	Rhizobiaceae	Agrobacterium	*A. tumefaciens*	*A. tumefaciens 1D1609*
R7A	Rhizobiaceae	Mesorhizobium	*M. loti*	*M. loti R7A*
LjRoot21	Xanthomonadaceae	Pseudoxanthomonas	-	-

Supplementary figure 1. **Bacterial strains used to create the SynCom19 inoculum for the scRNA-Seq experiment.** a) phylogenetic tree generated with recA full length DNA sequences. b) List of the isolates and taxonomies.

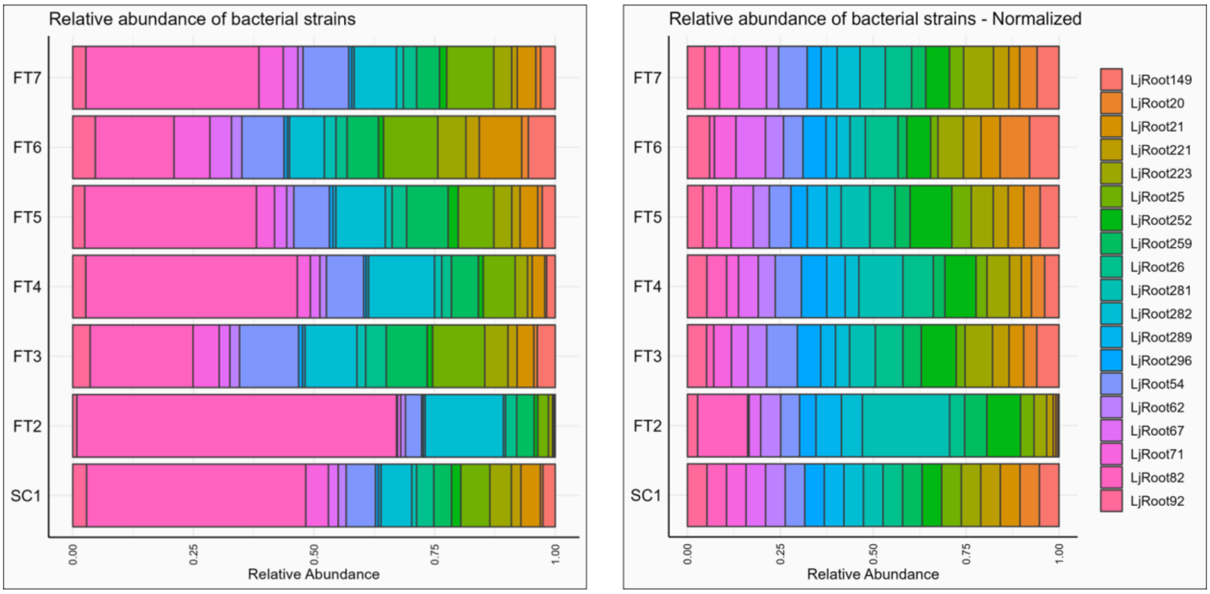

Supplementary figure 2. Stacked barplots representing the relative bacterial abundances of the SynCom19 strains. Colored bars FT2-7 represent rhizosphere samples harvested from the plates used to grow plants for single cell RNA-seq sequencing. SC1 is the input SynCom19 inoculum. a) Raw relative bacterial abundances b) Relative abundances normalized by the input SynCom19 inoculum.

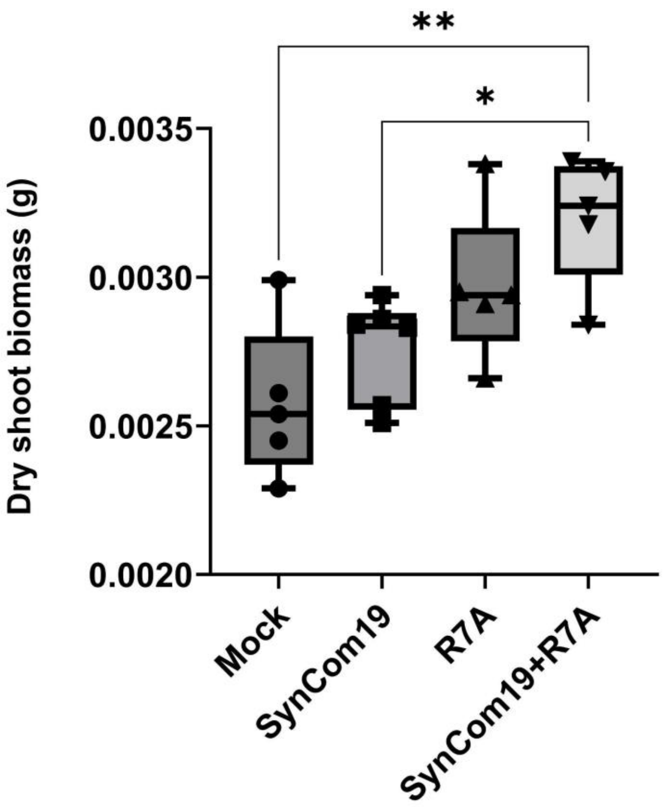

Supplementary figure 3. Effect of SynCom19, *M.loti* R7A and SynCom19 + M. loti R7A on *Lotus japonicus* shoot biomass. Dry shoot biomass of Lotus plants inoculated with SynCom19, M. loti R7A, or a combination of SynCom19 and R7A, compared to mock controls. Boxplots show the median, interquartile range, and minimum–maximum values; individual points represent biological replicates. Asterisks indicate statistical significance (*$p < 0.05$, **$p < 0.01$).

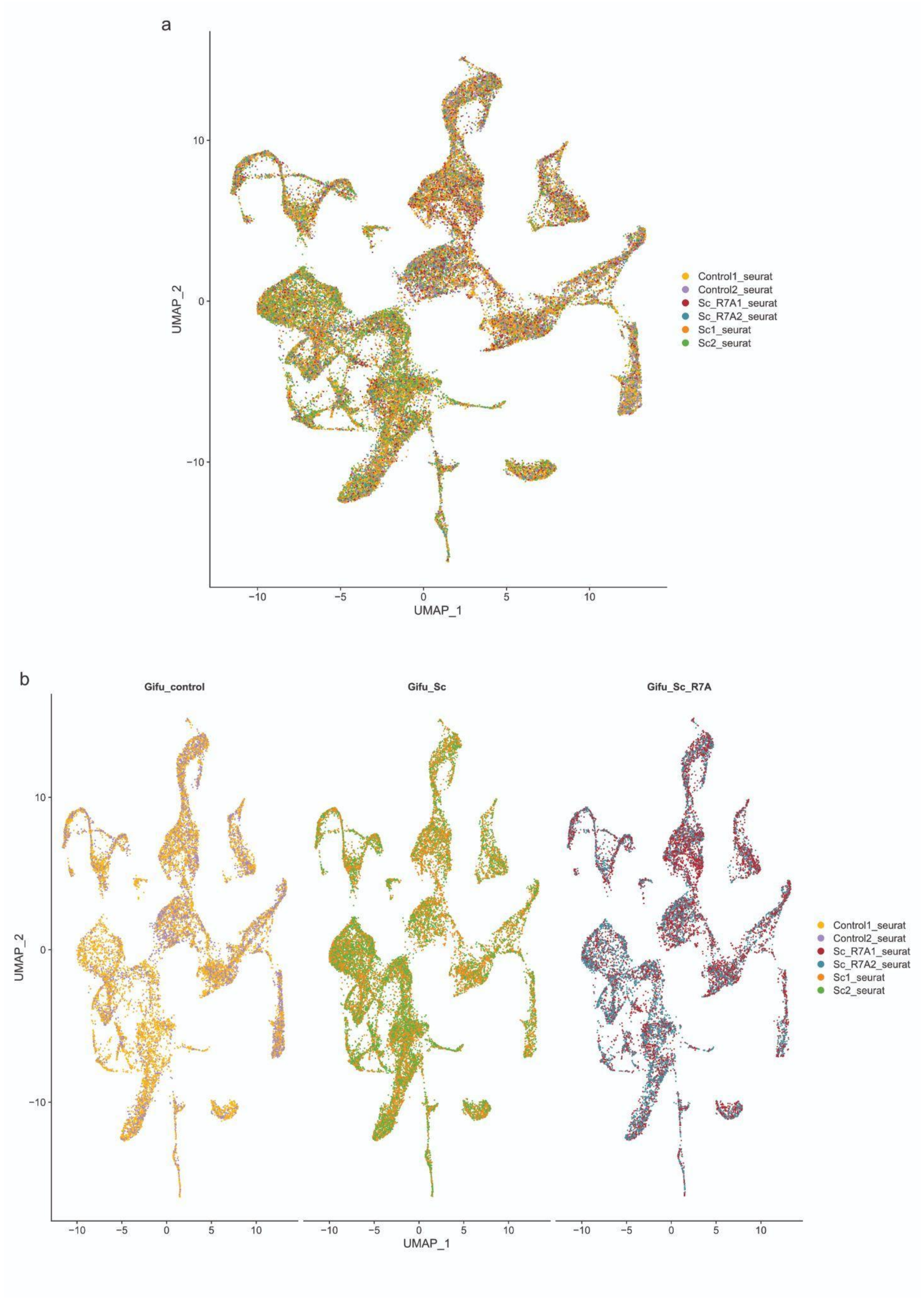

Supplementary figure 4. UMAP visualization of single cell transcriptomes from *Lotus japonicus* roots. a) UMAP plot showing the integrated single cell RNA-sequencing data set generated from roots harvested five days after inoculation with either a synthetic bacterial community (SynCom19) or the compatible symbiont *Mesorhizobium loti* R7A, alongside uninoculated controls. **b)** UMAP visualization under different inoculation treatments. Each point represents an individual protoplast-derived root cell and is colored according to the sample of origin (Control1, Control2, Sc_R7A1, Sc_R7A2, Sc1, Sc2). The UMAP embedding illustrates the global transcriptional relationships across all experimental conditions.

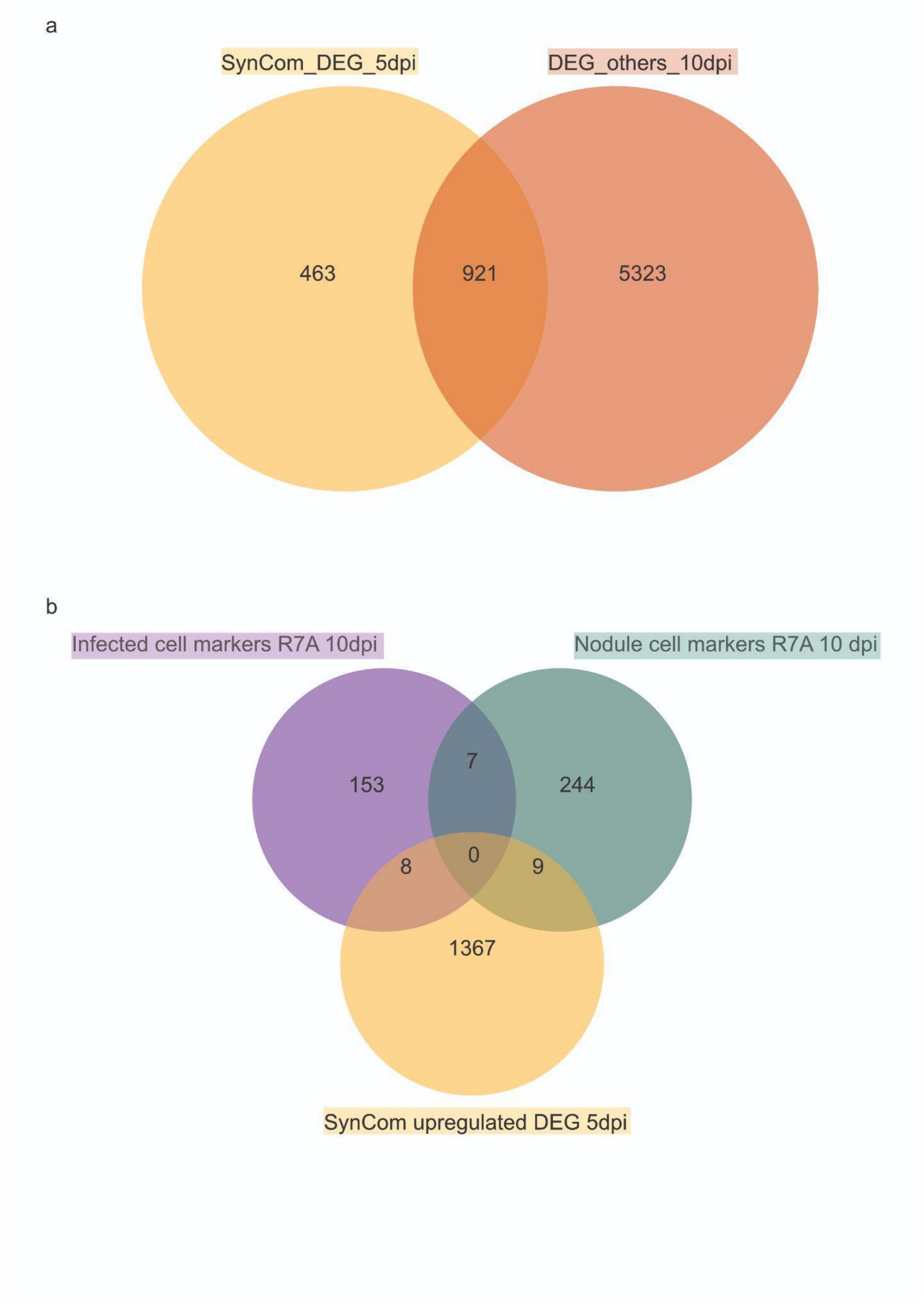

Supplementary figure 5. Overlaps in the transcriptional responses to symbiotic and commensal bacteria. a) Comparison of upregulated differentially expressed genes between SynCom19 at 5 dpi and R7A at 10 dpi alone in a previously published dataset[31] (Source Data "SynCom5dpi_DE_Genes" and "10_DE_Genes") b) Overlap between genes upregulated by SynCom19 at 5 dpi and the R7A-associated marker genes identified in a previous study[31] (Source Data "Infected_markers_10dpi" and "Nodule_markers_10dpi").

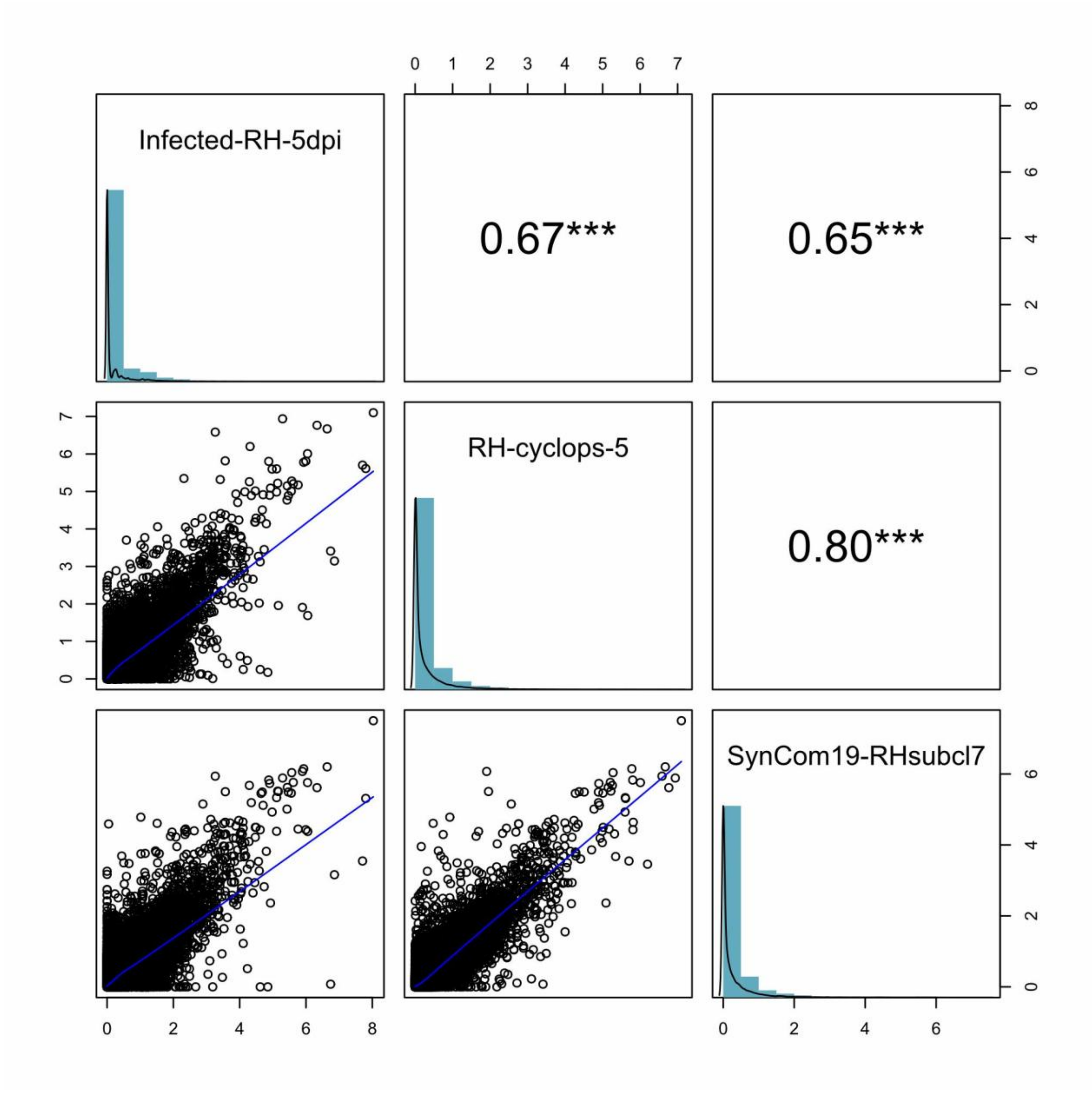

Supplementary Figure 6. Correlation analysis. Total gene expression across the three subpopulations and found a stronger correlation between SynCom19_RHsubcluster7 and RH_cyclops_5 subpopulations.

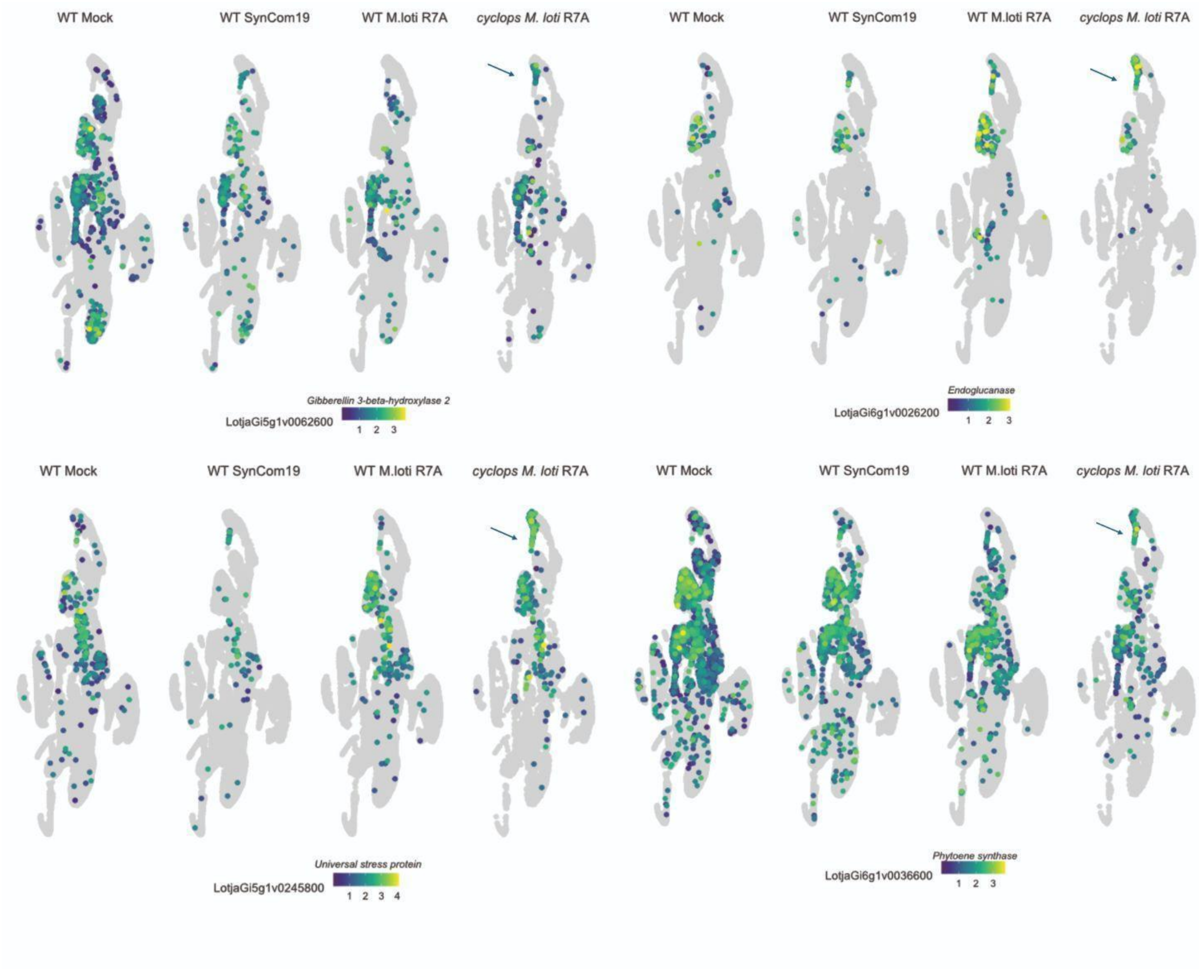

Supplementary figure 7. Expression patterns of selected genes commonly induced in a specific root hair population in WT SynCom19 and *cyclops M.loti* R7A.

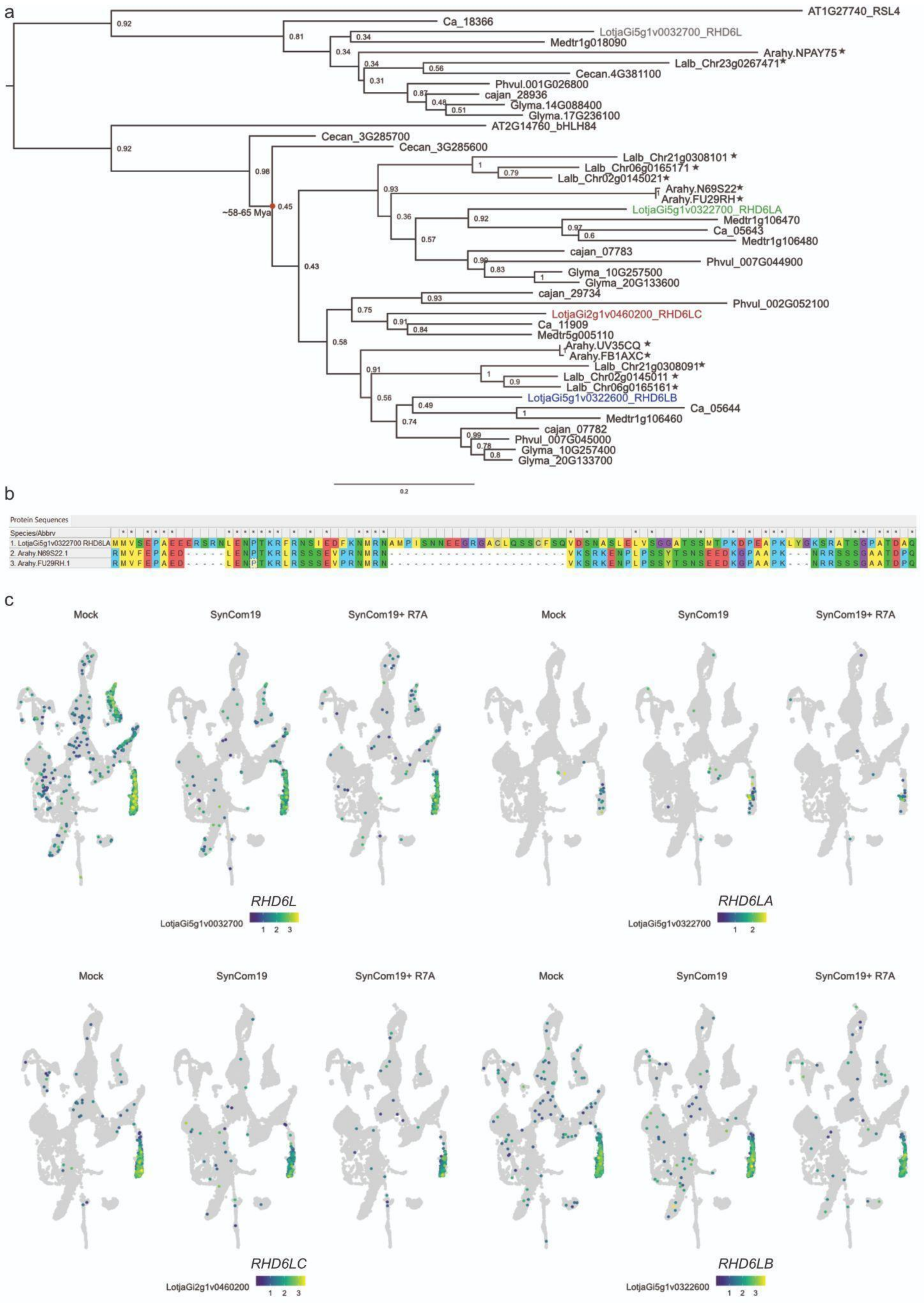
a
AT1G27740_RSL4
Ca_18366
LotjaGi5g1v0032700_RHD6L
Medtr1g018090
Arahy.NPAY75
Lalb_Chr23g0267471
Cecan.4G381100
Phvul.001G026800
cajan_28936
Glyma.14G088400
Glyma.17G236100
AT2G14760_bHLH84
Cecan_3G285700
Cecan_3G285600
Lalb_Chr21g0308101
Lalb_Chr06g0165171
Lalb_Chr02g0145021
Arahy.N69S22
Arahy.FU29RH
LotjaGi5g1v0322700_RHD6LA
Medtr1g106470
Ca_05643
Medtr1g106480
cajan_07783
Phvul_007G044900
Glyma_10G257500
Glyma_20G133600
cajan_29734
Phvul_002G052100
LotjaGi2g1v0460200_RHD6LC
Ca_11909
Medtr5g005110
Arahy.UV35CQ
Arahy.FB1AXC
Lalb_Chr21g0308091
Lalb_Chr02g0145011
Lalb_Chr06g0165161
LotjaGi5g1v0322600_RHD6LB
Ca_05644
Medtr1g106460
cajan_07782
Phvul_007G045000
Glyma_10G257400
Glyma_20G133700
~58-65 Mya
0.2
b
Protein Sequences
Species/Abbrv
1. LotjaGi5g1v0322700 RHD6LA
2. Arahy.N69S22.1
3. Arahy.FU29RH.1
c
Mock
SynCom19
SynCom19+ R7A
RHD6L
LotjaGi5g1v0032700
RHD6LA
LotjaGi5g1v0322700
RHD6LC
LotjaGi2g1v0460200
RHD6LB
LotjaGi5g1v0322600

Supplementary Figure 8. Evolution and expression of the RHD6L family. a) Maximum Likelihood phylogenetic tree (500 bootstrap replicates; node support values shown) based on protein sequence alignments generated with MUSCLE. *Lotus japonicus* proteins are highlighted in distinct colors. Stars indicate species known to perform intercellular or hybrid infection processes (*Arachis hypogaea*, *Lupinus albus*). *Cercis canadensis* (Ceca) is shown as an early-diverging lineage marking the split between Cercidoideae and Faboideae, estimated at 58-65 million years ago (red circle). **b)** Protein sequence alignment snapshot (LjRHD6LA, Arahy.RHD6LA_copy1, Arahy.RHD6LA_copy2) showing three conserved gaps located within the core of the "Transcription Factor bHLH83-related" domain (PTHR16223; amino acids 81-309). **c)** UMAP plots illustrating single-cell expression patterns of the four *L. japonicus RHD6L*-like paralogs. The ancestral *RHD6L* is broadly expressed across tissues, with enrichment in root hairs (RH) and root caps. *RHD6LA* shows specific expression in a subset of RH cells upon SynCom19 inoculation. *RHD6LB* and *RHD6LC* display strong expressions restricted to RHs.
