# Supplementary material for: RHD6LA regulates root hair responses to both symbionts and commensals
Source: Nat Commun. 2026 Mar 10;17:4447. doi: 10.1038/s41467-026-70504-1 (PMC13183932; doi:10.1038/s41467-026-70504-1)
Supplement: Supplementary file 2 — Description of Additional Supplementary Files [file 41467_2026_70504_MOESM2_ESM.pdf]

Description of Additional Supplementary Files

File name: Supplementary Data 1

Description: Data frame of all cell-level metadata (counts, dimensional reductions, cluster assignments, etc.).

File name: Supplementary Data 2

Description: The number of cells in each cluster.

File name: Supplementary Data 3

Description: Cluster-specific SynCom19 response.

File name: Supplementary Data 4

Description: Cluster-specific SynCom19 + *M. loti* R7A response.

File name: Supplementary Data 5

Description: Markers for cluster 59 (TopoMetry) in the SynCom19 + R7A treatment.

File name: Supplementary Data 6

Description: Markers for cluster 59 (TopoMetry) in the Mock treatment.

File name: Supplementary Data 7

Description: Markers for cluster 59 (TopoMetry) in the SynCom19 treatment. p_adj≤0.05 and pct.2≤0.02.

File name: Supplementary Data 8

Description: Infected genes 10dpi. p_adj≤0.05.

File name: Supplementary Data 9

Description: Cluster-specific infection response. p_adj≤0.05 and pct.2≤0.02

File name: Supplementary Data 10

Description: Nodule cell markers (see reference for definition).

File name: Supplementary Data 11

Description: Commonly induced genes in the 5dpi SynCom19 scRNA-seq dataset and in the 10dpi dataset of Lotus wild-type inoculated with M. loti R7A *.

File name: Supplementary Data 12

Description: Subcluster 7 from re-clustered Mock and SynCom19 root hair cells. p_val_adj≤0.05 and pct.2≤0.01.

File name: Supplementary Data 13

Description: Markers for 5dpi RH subcluster 6 WT *M. loti*. p_val_adj≤0.05 and pct.2≤0.01.

File name: Supplementary Data 14

Description: Markers for 5 dpi RH subcluster 5 *cyclops M. loti R7A*.

File name: Supplementary Data 15

Description: Commonly induced genes in the 3 populations of RH: "SynCom19_RHsubcl7", "Infected_RH_5dpi" and "RH_cyclops_5".

File name: Supplementary Data 16

Description: Cell ranger count summary.

File name: Supplementary Data 17

Description: Primers used for the 16s amplicon sequencing to amplify the V5-V7 region.

---

p_adj: adjusted p-value

pct2: percentage of other cells. where the gene is expressed

*Frank, M., Fechete, L.I. et al. Single-cell analysis identifies genes facilitating rhizobium infection in Lotus japonicus. Nat Commun 14, 7171 (2023)
