# Supplementary material for: RHD6LA regulates root hair responses to both symbionts and commensals
Source: Nat Commun. 2026 Mar 10;17:4447. doi: 10.1038/s41467-026-70504-1 (PMC13183932; doi:10.1038/s41467-026-70504-1)
Supplement: Supplementary file 4 — Reporting Summary [file 41467_2026_70504_MOESM4_ESM.pdf]

nature portfolio

Corresponding author(s): Stig Uggerhøj Andersen, Francesca Tedeschi

Last updated by author(s): 11/02/2026

Reporting Summary

Nature Portfolio wishes to improve the reproducibility of the work that we publish. This form provides structure for consistency and transparency in reporting. For further information on Nature Portfolio policies, see our Editorial Policies and the Editorial Policy Checklist.

Statistics

For all statistical analyses, confirm that the following items are present in the figure legend, table legend, main text, or Methods section.

n/a	Confirmed	
☐	☒	The exact sample size (*n*) for each experimental group/condition, given as a discrete number and unit of measurement
☐	☒	A statement on whether measurements were taken from distinct samples or whether the same sample was measured repeatedly
☐	☒	The statistical test(s) used AND whether they are one- or two-sided<br>*Only common tests should be described solely by name; describe more complex techniques in the Methods section.*
☐	☒	A description of all covariates tested
☐	☒	A description of any assumptions or corrections, such as tests of normality and adjustment for multiple comparisons
☐	☒	A full description of the statistical parameters including central tendency (e.g. means) or other basic estimates (e.g. regression coefficient) AND variation (e.g. standard deviation) or associated estimates of uncertainty (e.g. confidence intervals)
☐	☒	For null hypothesis testing, the test statistic (e.g. *F*, *t*, *r*) with confidence intervals, effect sizes, degrees of freedom and *P* value noted<br>*Give P values as exact values whenever suitable.*
☒	☐	For Bayesian analysis, information on the choice of priors and Markov chain Monte Carlo settings
☒	☐	For hierarchical and complex designs, identification of the appropriate level for tests and full reporting of outcomes
☐	☒	Estimates of effect sizes (e.g. Cohen's *d*, Pearson's *r*), indicating how they were calculated

Our web collection on statistics for biologists contains articles on many of the points above.

Software and code

Policy information about availability of computer code

Data collection	n/a
Data analysis	Microsoft® Excel® for Microsoft 365 MSO (Version 2505 Build 16.0.18827.20102) 64-bit, Cell Ranger v6.1.2 , Seurat 4.0.5, RStudio version 2023.06.0.421, GraphPad Prism 10, MUSCLE, Affinity Designer

For manuscripts utilizing custom algorithms or software that are central to the research but not yet described in published literature, software must be made available to editors and reviewers. We strongly encourage code deposition in a community repository (e.g. GitHub). See the Nature Portfolio guidelines for submitting code & software for further information.

Data

Policy information about availability of data

All manuscripts must include a data availability statement. This statement should provide the following information, where applicable:

- Accession codes, unique identifiers, or web links for publicly available datasets
- A description of any restrictions on data availability
- For clinical datasets or third party data, please ensure that the statement adheres to our policy

Supplementary Data is provided. UMAPs and gene expression data can be browsed at https://lotussinglecell.shinyapps.io/lotus_japonicus_single_cell_microbiome/. Raw data is available under ENA project accession PRJEB89066. The code used for single cell analysis, subclustering, 16S amplicon sequencing, DEGs, correlation analysis and GO term enrichment analysis can be found at DOI: 10.5281/zenodo.18598525
. The RDS files have been uploaded to FigShare https://doi.org/10.6084/m9.figshare.30271879.

Research involving human participants, their data, or biological material

Policy information about studies with human participants or human data. See also policy information about sex, gender (identity/presentation), and sexual orientation and race, ethnicity and racism.

Reporting on sex and gender	n/a
Reporting on race, ethnicity, or other socially relevant groupings	n/a
Population characteristics	n/a
Recruitment	n/a
Ethics oversight	n/a

Note that full information on the approval of the study protocol must also be provided in the manuscript.

Field-specific reporting

Please select the one below that is the best fit for your research. If you are not sure, read the appropriate sections before making your selection.

☒ Life sciences ☐ Behavioural & social sciences ☐ Ecological, evolutionary & environmental sciences

For a reference copy of the document with all sections, see nature.com/documents/nr-reporting-summary-flat.pdf

Life sciences study design

All studies must disclose on these points even when the disclosure is negative.

Sample size	For the scRNA-seq: Gifu SynCom: 100 whole roots in duplicate Gifu SynCom + M. loti (R7A): 100 whole roots in duplicate Gifu Mock: 100 whole roots in duplicate<br>Sample sizes for other experiments are given in figure legends and were chosen according to common standards in the field.
Data exclusions	No data were excluded
Replication	Experiments were repeated 2 to 3 times, except for scRNA-seq experiment
Randomization	No randomization was applied, however seedlings from different genotypes were grown under the same conditions.
Blinding	No blinding was performed

Reporting for specific materials, systems and methods

We require information from authors about some types of materials, experimental systems and methods used in many studies. Here, indicate whether each material, system or method listed is relevant to your study. If you are not sure if a list item applies to your research, read the appropriate section before selecting a response.

Materials & experimental systems

n/a	Involved in the study
x	Antibodies
x	Eukaryotic cell lines
x	Palaeontology and archaeology
x	Animals and other organisms
x	Clinical data
x	Dual use research of concern
	x Plants

Methods

n/a	Involved in the study
x	ChIP-seq
x	Flow cytometry
x	MRI-based neuroimaging

Dual use research of concern

Policy information about dual use research of concern

Hazards

Could the accidental, deliberate or reckless misuse of agents or technologies generated in the work, or the application of information presented in the manuscript, pose a threat to:

No	Yes
x	Public health
x	National security
x	Crops and/or livestock
x	Ecosystems
x	Any other significant area

Experiments of concern

Does the work involve any of these experiments of concern:

No	Yes
x	Demonstrate how to render a vaccine ineffective
x	Confer resistance to therapeutically useful antibiotics or antiviral agents
x	Enhance the virulence of a pathogen or render a nonpathogen virulent
x	Increase transmissibility of a pathogen
x	Alter the host range of a pathogen
x	Enable evasion of diagnostic/detection modalities
x	Enable the weaponization of a biological agent or toxin
x	Any other potentially harmful combination of experiments and agents

Plants

Seed stocks	Both wild-type and LORE1 mutant (https://lotus.au.dk/lore1/search) Lotus japonicus seeds used in the experiments were propagated in the greenhouse at Aarhus University, located at Hvidbjergvej 21, Ølsted, 8380 Trige
Novel plant genotypes	LORE1 insertion mutants : Genotyping by PCR (Phire kit for genotyping) has been performed on a segregant mutant population, 4 homozygous plants have been isolated for each allele to produce seeds.
Authentication	n/a
